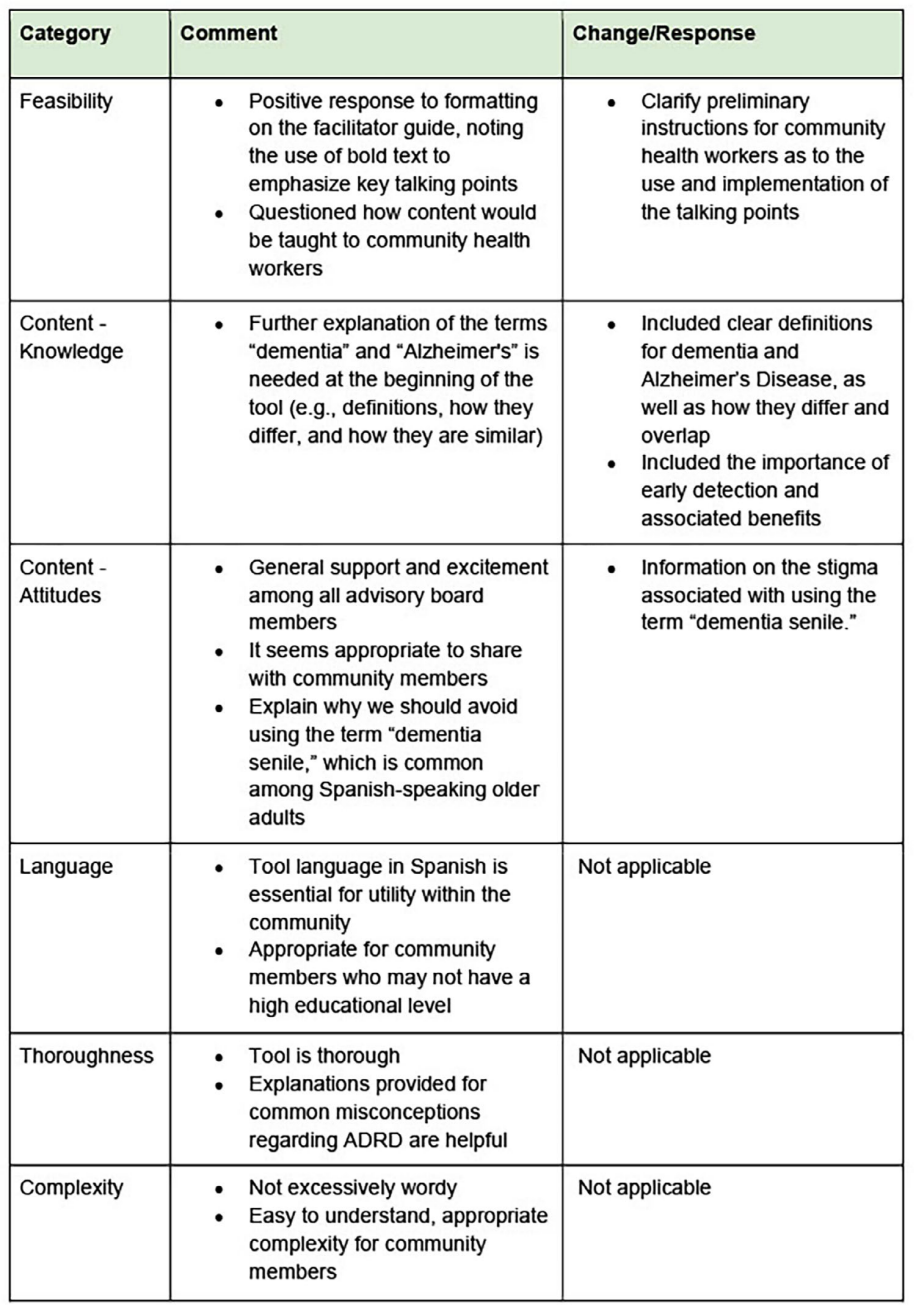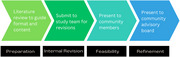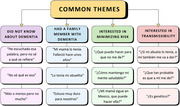# Development of an Educational Tool to Reduce Misconceptions about Alzheimer's Disease and Related Dementias in the Latine Population

**DOI:** 10.1002/alz70860_097576

**Published:** 2025-12-23

**Authors:** Rodrigo G Delatorre, Maria del Carmen Rosales, Maria C Mora Pinzon

**Affiliations:** ^1^ University of Wisconsin School of Medicine and Public Health, Madison, WI, USA; ^2^ Department of Medicine, Division of Geriatrics, School of Medicine and Public Health, University of Wisconsin‐Madison, Madison, WI, USA

## Abstract

**Background:**

Despite a higher prevalence of Alzheimer's disease and related dementias (ADRD), Latine individuals face higher rates of underdiagnosis and misdiagnosis. Furthermore, limited English proficiency and low socioeconomic status are associated with reduced physician service utilization, further delaying diagnosis. A community‐based approach has been suggested to increase awareness of ADRD which in turn will improve healthcare seeking behaviors and subsequently increase diagnosis and evidence‐based management. This study aims to describe the development and assessment of the feasibility and acceptability of a community‐based educational tool to increase ADRD awareness among Spanish‐speaking Latine individuals.

**Method:**

This tool was designed to be used by non‐healthcare professionals (e.g., community health workers) and consisted of a one‐page document that addressed the common beliefs and misconceptions, using a question‐and‐answer approach to serve as a guide for conversations with Latine individuals. The development of this tool required several steps, including conducting a literature review, identifying knowledge gaps and misconceptions, and creating the initial draft, feasibility assessment, and refinement (Figure 1). All the documents were developed in Spanish. A draft of the tool was piloted at a community health fair. Comments and interactions with attendees at the health fair and feedback from a Latine community advisory board were used to refine the tool. No personal information was collected from individuals. Notes and memos were used to collect information and revised in a team setting to guide changes in the tool.

**Result:**

Community members attending the health fair expressed a strong willingness to engage in discussions about dementia, regardless of age or gender. One of the most common questions were on the distinctions between Alzheimer's disease and dementia, as well as the heritability and modifiable risk factors for dementia (Figure 2). The community advisory board considered the educational tool to be useful and acceptable (Table 1).

**Conclusion:**

A community‐based educational tool developed with input from community members and advisory boards, has the potential to increase engagement and adoption within the Latine community. Future pilot studies will assess the effectiveness of this intervention in promoting accurate medical knowledge about dementia and facilitating timely memory screenings.